# Getting better with age: Lessons from the Kenya Long‐term Exclosure Experiment (KLEE)

**DOI:** 10.1111/ele.14466

**Published:** 2024-12-31

**Authors:** Corinna Riginos, Duncan M. Kimuyu, Kari E. Veblen, Lauren M. Porensky, Wilfred O. Odadi, Ryan L. Sensenig, Harry B. M. Wells, Truman P. Young

**Affiliations:** ^1^ The Nature Conservancy Lander Wyoming USA; ^2^ Department of Natural Resources Karatina University Karatina Kenya; ^3^ Mpala Research Centre Nanyuki Kenya; ^4^ Department of Wildland Resources and Ecology Center Utah State University Logan Utah USA; ^5^ USDA‐ARS Rangeland Resources and Systems Research Unit Fort Collins Colorado USA; ^6^ Department of Natural Resources Egerton University Egerton Kenya; ^7^ Department of Biology University of Notre Dame South Bend Indiana USA; ^8^ Department of Ecology and Evolutionary Biology Princeton University Princeton New Jersey USA; ^9^ Lolldaiga Hills Research Programme Nanyuki Kenya; ^10^ Department of Plant Sciences University of California Davis California USA; ^11^ Graduate Degree Program in Ecology Colorado State University Fort Collins Colorado USA

**Keywords:** delayed responses, drought, environmental change, herbivore exclosures, livestock, mega‐herbivores, year effects

## Abstract

The Kenya long‐term exclosure experiment (KLEE) was established in 1995 in semi‐arid savanna rangeland to examine the separate and combined effects of livestock, wildlife and megaherbivores on their shared environment. The long‐term nature of this experiment has allowed us to measure these effects and address questions of stability and resilience in the context of multiple drought‐rainy cycles. Here we outline lessons learned over the last 29 years, and how these inform a fundamental tension in long‐term studies: how to balance the need for question‐driven research with the intangible conviction that long‐term data will yield valuable findings. We highlight the value of (1) identifying experimental effects that take many years to manifest, (2) quantifying the effects of different years (including droughts) and (3) capturing the signatures of anthropogenic change. We also highlight the potential for long‐term studies to create a collaborative community of scientists that brings new questions and motivates continued long‐term study.

## Graphical abstract

The Kenya Long‐term Exclosure Experiment (KLEE) was established in 1995 in a semi‐arid savanna rangeland to examine the separate and combined effects of livestock, wildlife, megaherbivores and fire on each other and on their shared environment. Illustrated here are some of the many relationships experimentally demonstrate from KLEE.

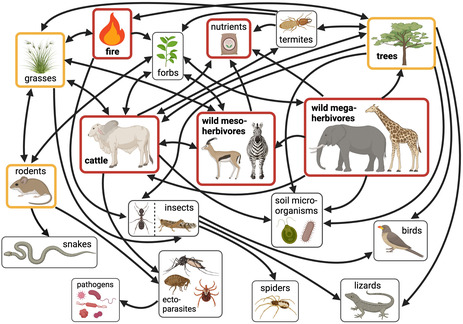

## LONG‐TERM ORIGINS

The Kenya Long‐term Exclusion Experiment (KLEE) was established in 1995, but its origins are traced to the 1970s when one of TPY's mentors gave this advice: start a long‐term project. It need not be strongly conceptual or experimental; just pick something, start monitoring it and keep monitoring. The wisdom of this advice is justified: the longest‐duration ecological projects have been some of the most fruitful, particularly in a world experiencing global change (e.g., Campbell et al., 2022; Inouye & Barr, 2006; Krebs et al., 2023; Likens, 1989; Lindenmayer et al., 2012; Prather et al., 2023; Reinke et al., 2019; Risser, 1991).

Following this suggestion, however, requires a certain faith that as‐yet unanticipated valuable findings will come from long‐term studies, juxtaposed against the reality that funding cycles are often shorter‐term and demand conceptually based predictions. How should researchers and funders navigate this tension? Here, we explore insights that have come out of nearly three decades of the KLEE project.

**FIGURE 1:**
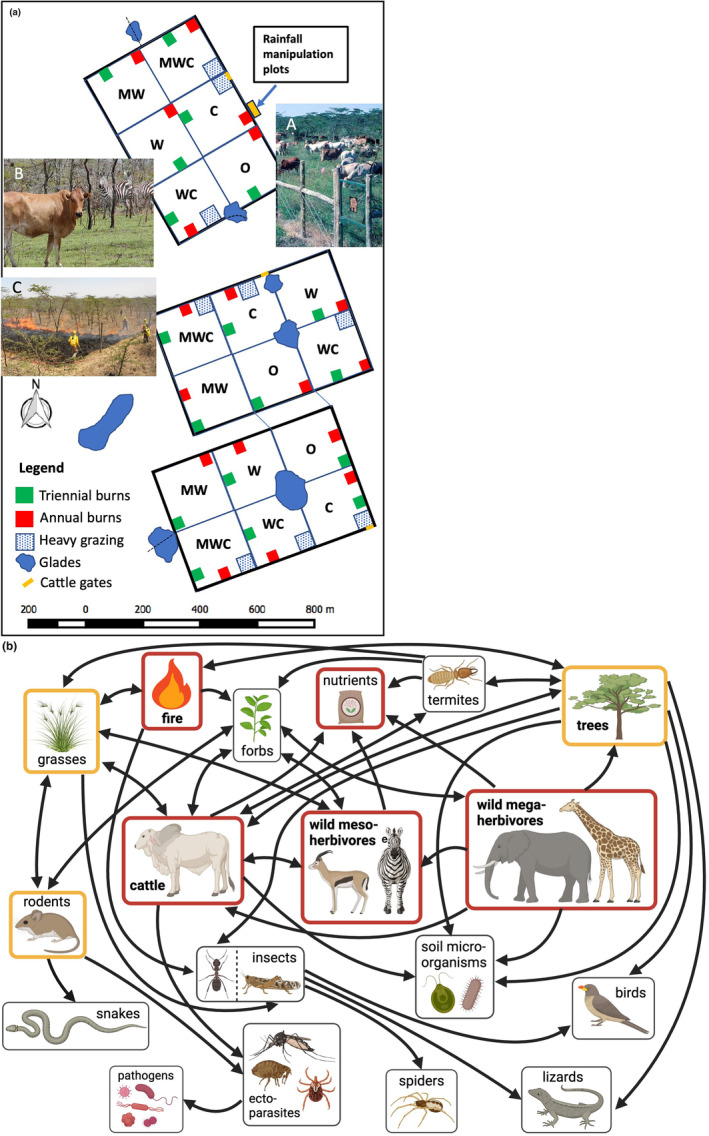
(a) Schematic of the Kenya Long‐term Exclosure Experiment (KLEE), showing the layout of the different herbivore treatment plots (using semi‐permeable barriers) and the multiple embedded experiments. The letters inside each plot represent the (combinations of) large mammalian herbivores allowed access: C, cattle; W, meso‐herbivores (25–800 kg); M, megaherbivores (>1000 kg); O, no large herbivores allowed. ‘Glades’ are treeless anthropogenic patches arising from long‐abandoned cattle enclosures (‘bomas’). Photos illustrate (A) cattle grazing in a C plot, with entry gate in lower right, (B) cattle and plains zebras in a recently burned WC plot, (C) KLEE crew conducting a controlled burn in one of the plots. (b) Some of the many relationships demonstrated and inferred from KLEE. Variables in red and orange boxes have been experimentally manipulated (as well as being measured as response variables). Red boxes are experimental manipulations fully embedded in and crossed with the KLEE herbivore treatments; orange boxes are separate replicated experimental manipulations associated with KLEE. Arrows indicate the direction of each demonstrated effect. Not all relationships have been represented here. For example, termite mounds strongly modify plant communities and provide refuges and stable burrows for both snakes and rodents. Interactive effects are not illustrated, like the fact the elephants mitigate the effects of cattle on other ecosystem variables (Young et al., 2022). Also not included here are the rich set of experimental and descriptive studies of anthropogenic glades associated with KLEE (Porensky & Veblen, 2012, 2015; Veblen, 2012; Veblen & Porensky, 2019; Veblen & Young, 2012). This figure was updated and adapted from Young et al., 2018.

**FIGURE 2:**
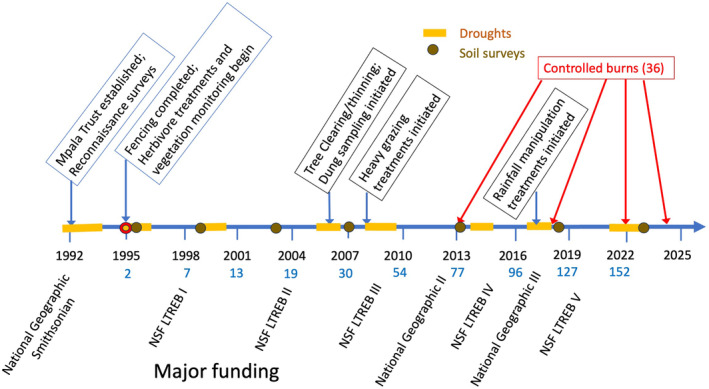
Timeline of the Kenya Long‐term Exclosure Experiment (KLEE), highlighting some key developments and funding (the latter does not include multiple NSF GRF, DDIG, REU and ROU grants). Herbivore treatments include separate and combined exclusions of meso‐herbivores, mega‐herbivores, cattle and rodents. Dung counts are carried out to estimate herbivore use of different treatments. Drought events are highlighted in orange. Brown dots are soil surveys. An adjacent rainfall manipulation experiment was initiated in 2017. The numbers in blue beneath each date are the cumulative numbers of peer‐reviewed publications from the project. These totals do not include 33 outreach publications or 30 American and Kenyan graduate dissertations.

The Kenya Long‐term Exclosure Experiment is predicated on the conceptual understanding that rainfall, wild herbivores, livestock and fire are dominant within‐site forces in both short‐term and long‐term savanna dynamics (in addition to across‐site effects of climate and soils). The experiment is composed of three blocks of six 4‐ha plots assigned to different treatment combinations of large mammalian herbivores (Figure 1). The study was initiated with specific questions about competition and coexistence between different wild and domestic herbivore guilds and their separate and combined effects on the community (Young et al., 1997). Time and layering of additional manipulations (Figure 2) have contributed to our understanding of how herbivore guilds affect community ecology, trophic dynamics, species coexistence, disease ecology, tree–grass interactions, fire ecology, mutualisms, spatial heterogeneity and other core topics in ecology (Figure 1b; Data S1).

Below, we present three ways that KLEE exemplifies the value of long‐term studies and has yielded payoffs far beyond the original scope of purpose and predictions. We then discuss navigating the balance between shorter‐term inquiry and longer‐term outcomes, how collaborations promote success, and how and when long‐term studies are likely to be most valuable.

## REASON FOR FAITH: THE (SOMETIMES UNANTICIPATED) VALUE OF LONG‐TERM DATA

### Delayed and unexpected findings

Within the first few years of KLEE, there was clear evidence for the predicted competition between cattle and wild large and small herbivores (Keesing, 2000; Young et al., 2005). Longer‐term study, through multiple drought cycles, reinforced evidence for competition during dry seasons but revealed unexpected facilitation between wildlife and cattle during wet seasons (Kimuyu et al., 2017; Odadi et al., 2011). Long‐term data also revealed, surprisingly, a degree of functional similarity in the effects of wildlife and moderately stocked (but not heavily stocked) cattle on plant community composition and primary productivity (Charles et al., 2017; Veblen et al., 2016; Wells, Crego, et al., 2022; Wells, Porensky, et al., 2022)—further suggesting conservation opportunities for landscapes shared by livestock and wildlife.

Herbaceous community composition did not differ significantly across treatments until 5–10 years into the experiment, whereupon it began to make pronounced shifts (Riginos et al., 2018; Veblen et al., 2016). Similarly, differences in soil chemistry across herbivore treatments only became apparent two decades into the experiment (Sitters et al., 2020). These delays are perhaps not surprising in a system dominated by perennials (Lv et al., 2023). Often, plant compositional shifts can take even longer (e.g., >30 years in shortgrass prairie; Porensky et al., 2017). With KLEE, we found that only some of the original core conceptual questions could be answered with shorter‐term data, whereas long‐term data have yielded more, and richer, answers—highlighting how long‐term experiments are necessary to capture effects that are slow to accumulate.

### Capturing oscillations and inflection points

Community ecology studies, including those within KLEE, set out to understand the relationships among species. Yet many, if not most, ecological processes vary substantially among years (often called ‘year effects’) due to temporally variable environmental and anthropogenic influences as well as internal dynamics (Vaughn & Young, 2010; Werner et al., 2020). Results from any one or few years generally provide an incomplete picture of ecological and evolutionary processes or the effects of intensifying global change and may be idiosyncratic.

Long‐term data through multiple periods of below‐ and above‐average rainfall in KLEE (Figure 2) have helped us to separate dynamics that are periodic but consistent versus those that are accelerating. For example, the competition–facilitation balances between cattle and wildlife (Kimuyu et al., 2017; Odadi et al., 2011) and between different grass species (Veblen & Young, 2012), the dynamics among herbivores, grasses and trees (Riginos, 2015), and tick responses to cattle presence (Keesing et al., 2018) are all contingent upon rainfall. In contrast to these oscillating patterns, we have also found that drought‐release years (the first rainy year after a drought) are important inflection points for directional—or accelerating—changes in plant community composition, contingent upon herbivore treatment (LaMalfa et al., 2021; Porensky et al., 2013; Riginos et al., 2018; Veblen et al., 2016). For example, successive post‐drought periods widen the dissimilarity among treatments in community composition over time via rapid increases in certain grasses, at the expense of other plants (Riginos et al., 2018).

Long‐term data from KLEE have helped to verify oscillation and inflection dynamics, as we have seen both of these patterns play out repeatedly. In a future with more extreme rainfall dynamics, long‐term data may reveal where these patterns hold or break—for example, we may see that more prolonged droughts lead more to inflection patterns rather than to oscillations or resilience.

### Long‐term system change

The Kenya Long‐term Exclosure Experiment was designed in part to improve our understanding of how to manage a complex system with multiple land‐use objectives—specifically, livestock husbandry and wildlife conservation (Young et al., 2018, 2021). Long‐term studies like KLEE can also capture—and need to cope with—shifting sociological and ecological baselines in an era of rapid change.

An initial motivation for KLEE was to study ecological dynamics outside of parks and protected areas that included livestock grazing, declining wildlife numbers, fire suppression and woody encroachment (du Toit & Cumming, 1999; Reid, 2012). The addition of a heavily grazed treatment within each cattle plot and an adjacent tree‐thinning and clearing experiment in 2008 were adjustments to understand better emerging regional trends of heavy livestock grazing (Wells, Crego, et al., 2022; Wells, Porensky, et al., 2022) and woody losses to elephants and/or charcoal production (Riginos et al., 2009). The addition of on‐going fire treatments in 2014 has assisted in understanding the effects of fire inclusion as well as exclusion on plant and animal community dynamics (e.g., Masudi et al., 2024; Ngugi et al., 2022; Werner et al., 2021). As we enter the fourth decade of the study, KLEE is poised to capture the effects of additional system changes, most notably invasive species. Non‐native *Opuntia* cacti have spread (Wells et al., 2023), and pantropical invasive big‐headed ants (*Pheidole megacephala*) are projected to reach the KLEE plots soon, with potentially devastating effects on the ecosystem, as has been documented nearby (Hays et al., 2022). These changes, past and future, underscore the value of long‐term data; a rich and extensive baseline makes it possible to learn from and adapt to these changes as they occur.

## LONG‐TERM FUNDING AND THE PRESSURE FOR EXPANDING HORIZONS

The above examples illustrate the value of long‐term experiments, but also that many of the richest findings have arisen as surprises, or on unpredictable time horizons, out of shorter‐term questions. Funding long‐term experiments, even just to maintain infrastructure and core data collection, is a major challenge, and although the value of the data sets increases with each year, funders cannot support research simply on faith that valuable findings will eventually happen. This leads to an understandable—and both motivating and challenging—pressure from funders to come up with new questions and conceptual approaches, not merely to continue to monitor in the context of the original questions (c.f. Alber et al., 2021).

For KLEE, an effective approach in the first 10–15 years was to build funding proposals around shorter‐term questions that added dimensions—new species or guilds, or nested manipulations—to those previously studied. In the examples above, the long‐term value came out of pursuing shorter‐term studies and adding layers to them. Over time, the accumulated results have drawn in new collaborations that have expanded the project's scope as the field of ecology itself has evolved. For example, it is only now, with multiple rainy/drought cycles, that we can carry out powerful analyses of longer‐term ecosystem stability and resilience in the face of multiple interacting stressors (Ebel et al., 2022; Masudi et al., 2024)—a conceptual topic that is integral to recent KLEE inquiry and funding cycles.

## COLLABORATIVE FEEDBACKS

Although KLEE was inspired in part by simple advice to collect long‐term data, we believe that much of its effectiveness has stemmed from the more complex phenomenon of collaborative feedback. The unanticipated findings are not just ecological; they are driven by the diverse researchers and local community partners (such as community members, ranchers and field technicians)—each with their own unique ideas, new paradigms and novel skill sets—that have come to the project over several decades. The long‐term nature of KLEE has allowed a congregation of minds and insights to develop, yielding new synergies and lines of inquiry, which in turn have helped propel the project's evolution (c.f. Woolley et al., 2015). As one example (see also Data S1), within the first years of KLEE, there was a large increase in herbaceous biomass and an attendant doubling of rodent numbers in plots from which large herbivores had been excluded (Keesing, 1998, 2000). These early rodent results drew additional collaborators and formed the basis of a diverse set of studies examining disease vectors and pathogens (Keesing et al., 2013, 2018; McCauley et al., 2008; Tchouassi et al., 2021; Weinstein et al., 2017; Young et al., 2016)—contributing to the relatively new discipline of disease ecology in ways not anticipated at the start of the experiment. These kinds of novel collaborations and transdisciplinary workstreams are more likely to arise when there is an established study infrastructure and enough results to draw attention from new collaborators who, in turn, help to sustain the study and funding for long‐term work.

## CONCLUSION: LONG‐TERM STUDY AND DEEP UNDERSTANDING

Those deciding whether to embark on, or to continue, a long‐term study ideally consider both the value of the novel, focused questions associated with shorter‐term funding cycles as well as the intangible faith that other, valuable results will continue to emerge. From the KLEE experience, we suggest that long‐term studies will be most valuable when ecological cycles are long and/or response rates are slow, when inflection points continue to occur and/or treatments continue to diverge in interesting ways, when new drivers of ecosystem dynamics are anticipated, and when the study continues to draw in vibrant new perspectives, collaborators or opportunities to test new methods. A periodic assessment of whether these are still happening may be needed to justify continuing a study (c.f. Cusser et al., 2021).

Lastly, we note that perhaps the greatest value of long‐term studies is the opportunity to get deeply immersed in a system, to learn it well enough to recognize even its more subtle patterns and departures from them. One could call this ‘a feeling for the ecosystem’. The authors are indebted not only to the duration of KLEE but also to the long‐term field assistants in developing the ‘feeling for’—and deep knowledge of—the ecosystem that has so greatly enriched the project. As suggested by TPY's mentor a half‐century ago, it is not merely spending time with a system, but also the act of consistently and systematically gathering information from it, that provides some of the most valuable insights. Not every study can or should be long term. Yet we should not undervalue the conviction that powerful insights will emerge when not just individuals, but an entire research community, are invited to consider a particular ecosystem, through multiple lenses, over multiple decades.

## AUTHOR CONTRIBUTIONS

CR and TPY contributed equally to the writing of the manuscript, and all authors contributed substantive ideas and revisions.

### PEER REVIEW

The peer review history for this article is available at https://www.webofscience.com/api/gateway/wos/peer‐review/10.1111/ele.14466.

## Supporting information


Data S1.

## Data Availability

Not new data reported here.
