# Supplementary material for: Getting better with age: Lessons from the Kenya Long‐term Exclosure Experiment (KLEE)
Source: Ecol Lett. 2024 Dec 31;27(12):e14466. doi: 10.1111/ele.14466 (PMC11686941; doi:10.1111/ele.14466)
Supplement: Supplementary file 1 — Data S1. [file ELE-27-0-s001.docx]

**Supplemental Material:**

***KLEE Publication Record and Collaborations***

Thus far (June 2024), the KLEE project has produced (and contributed to) 160 peer-review publications, 33 outreach publications, and 30 graduate dissertations, making this U.S./Kenya collaboration the most productive experiment ever carried out on the continent of Africa. It has supported the graduate research of 11 masters students and eight doctoral students in Kenya, three masters students and 12 doctoral students in the U.S. and Canada, and more than a dozen U.S. and Kenya undergraduate projects (for a list of publications and dissertations, see <https://tpyoung.ucdavis.edu/kleepubs>).

KLEE contributes to multiple long-term ecological databases: the LOng-Term Vegetation Sampling (LOTVS) database, and the Grazing Exclosure Database (GEx), and the Nutrient Network (NutNet). The latter two are explicitly experimental. Additional long-term (restoration) experiments from our lab are contributors to the Global Restore Project (GRP) and the Global Arid Zone Project (GAZP). These networks and collaborations are invaluable in allowing for insightful meta-analyses and syntheses, multiplying the power and increasing the generality of individual long-term studies (e.g., Koerner et al. 2018, Forbes et al. 2019, Valencia et al. 2020a,b, Shackleford et al. 2021, Buisson et al. 2021, Sperandi et al. 2022, Conti et al. 2023, Pringle et al. 2023, Karp et al. 2024).

In addition to the mega-authored papers from these collaborative databases, KLEE has hosted collaborators from the U.S., Kenya, South Africa, Canada, Netherlands, Belgium, Cameroon, Germany, Finland, Mexico, and Great Britain. These collaborations have been fostered in no small part by the collegial climate provided by the shared infrastructure of the Mpala Research Centre. We note that although some of these collaborations could have been completed in a shorter-term KLEE experiment, they never would have happened if there had not been time for the experiment to become known and draw the attention of other ecologists who had new ideas of things to test within KLEE.

Even before the recent mandates of funding sources and journals, KLEE was making its datasets publicly available, as a way of encouraging and facilitating collaboration.

Buisson, E., A. Fidelis, G.E. Overbeck, I.B. Schmidt, G. Durigan, T.P. Young, et al. (2021). A research agenda for the restoration of tropical and subtropical grasslands and savannas. *Restor. Ecol.,* 29(S1), e13292.

Conti, L., E. Valencia, T. Galland, L. Götzenberger, J. Leps, A. Vojtkó, et al. (2023). Functional traits trade-offs define plant population stability worldwide. *Proc. Roy. Soc. B*, 290, 20230344.

Forbes, E.S., J.H. Cushman, D.E. Burkepile, T.P. Young, M. Klope & H.S. Young. (2019). Synthesizing the effects of large, wild herbivores on ecosystem function. *Funct. Ecol.*, 33, 1597-1610.

Karp, A.T., S.I. Koerner, G.P. Hempson, J.O. Abraham, T.M. Anderson, W.J. Bond, et al. (2024). Grazing herbivores reduce herbaceous biomass and fire activity across broad-scale savanna gradients. *Ecology Letters*, 27, e14450.

Koerner, S.E**.**, M.D. Smith, D.E. Burkepile, N. Hanan, M.L. Avolio, S.L. Collings, et al. (2018). Resolving variation in herbivore effects on plant biodiversity – change in dominance as a global mechanism. *Nature Ecol. & Evol.*, 2, 1925-1932.

Pringle, R.M., J.O. Abraham, T.M. Anderson, T.C. Coverdale, A.B. Davies, C.L. Dutton, et al. (2023). Impacts of large herbivores on terrestrial ecosystems. *Current Biology*, 33, 584-610.

Shackelford, N., G.B. Paterno, D.E. Winkler, T.E. Erickson, E.A. Leger, L.N. Svejcar, et al. (2021). Drivers of seedling establishment success in dryland restoration efforts. *Nature Ecol. & Evol.*, 5, 1283-1290.

Sperandii, M.G., F. de Bello, E. Valencia, L. Götzenberger, M. Bazzichetto, T. Galland, et al. (2022). LOTVS: a global collection of permanent vegetation plots. *J. Veg. Sci.,* 25, e13115.

Valencia, E., F. de Bello, T. Galland, P.B. Adler, J. Lepš, A. E-Vojtkó, et al. (2020a). Synchrony matters more than species richness in plant community stability at a global scale. *PNAS*, 117, 24345–24351.

Valencia, E., F. de Bello, J. Lepš, T. Galland, A. E-Vojtko, L. Conti et al. (2020b). Directional trends in species abundance over time can lead to a widespread overestimation of asynchrony. *J. Veg. Sci.*, 31, 792– 802.
